# The Satisfaction with Life Scale and the Subjective Well-Being Inventory in the General Korean Population: Psychometric Properties and Normative Data

**DOI:** 10.3390/ijerph16091538

**Published:** 2019-04-30

**Authors:** Young Ho Yun, Ye Eun Rhee, Eunkyo Kang, Jin-ah Sim

**Affiliations:** 1 Department of Biomedical Science, Seoul National University College of Medicine, Seoul 03080, Korea; graceyeun@gmail.com (Y.E.R.); jinah811@gmail.com (J.-a.S.); 2 Department of Biomedical Informatics, Seoul National University College of Medicine, Seoul 03080, Korea; 3 Department of Family Medicine, Seoul National University Hospital, Seoul 03080, Korea; ekherb@naver.com; 4 Cancer Research Institute, Seoul National University, Seoul 03080, Korea

**Keywords:** Satisfaction with Life Scale (SWLS), Subjective Well-Being Inventory (SWBI), Korea, Subjective Well-Being (SWB), Satisfaction with Life (SWL)

## Abstract

This study aims to evaluate the psychometric properties of the Satisfaction with Life Scale (SWLS) and the Subjective Well-Being Inventory (SWBI) in a nationally representative sample in Korea. A total of 1200 people completed the semi-structured, self-reported questionnaire, which included five items from the SWLS and 14 items from the SWBI. All items and the total score of both the SWLS and the SWBI showed high internal consistency (with Cronbach’s alphas of 0.886 and 0.946, respectively). The item-total correlation values for both measures were in the ranges of 0.71–0.75 and 0.65–0.80, respectively. There were positive correlations between the SWLS and SWBI (*r* = 0.59, *p* = 0.01). The SWLS, SWBI and global well-being (GWB) scores were positively correlated with the McGill Quality of Life subscales (*p* = 0.01) but negatively correlated with the Patient Health Questionnaire-9 (*p* = 0.01). Participants under 50 years old (adjusted odds ratio [aOR] = 1.30, 95% confidence interval [CI] = 1.00–1.69) and those in rural areas (aOR = 1.63, 95% CI = 1.28–2.07) had higher scores on the SWLS than other participant groups. Participants who were under 50 years old (aOR = 1.47, 95% CI = 1.12–1.92), were male (aOR = 1.33, 95% CI = 1.04–1.71), were married (aOR = 1.51, 95% CI = 1.13–2.01), lived in rural areas (aOR = 2.30, 95% CI = 1.35–3.91), or had higher incomes (aOR = 1.30, 95% CI = 1.02–1.65) showed higher SWBI scores. This study showed that the SLWS and SWBI have good psychometric properties and could be applicable to Korea.

## 1. Introduction

Systematic tracking of subjective well-being (SWB) at both the individual and national levels could give policy-makers and governments useful information for monitoring the positive effects of public assistance programs and improving societies in areas beyond economic development [1,2]. SWB is defined as people’s evaluation of their lives, including positive emotion, engagement, satisfaction, and meaning [3,4]. In particular, a substantial amount of evidence supports the conclusion that high SWB is generally beneficial to health and longevity, productivity, and social relationships [5,6,7,8]. Although economics plays a critical role in policy decisions, there is only a small association between national economics and human happiness. [7,9]. Consequently, SWB is a very important part of quality of life.

The American psychologist Ed Diener proposed that nations should adopt and use SWB as a social indicator that reflects the quality of life in countries in conjunction with national accounts of the economy [10]. Following this proposal, several prestigious scientific and international organizations have also recommended the creation of such national accounts. For example, the Organization of Economic Cooperation and Development (OECD) issued guidelines for national measures of SWB, and more than 40 countries have adopted national measures of SWB [1].

Over the last 30 years, the science of well-being has shown that measures of SWB are valid and reliable [11]. SWB includes an affective component—that is, it measures both pleasant and unpleasant affect—as well as a cognitive component that refers to life satisfaction as a conscious cognitive judgment [12,13,14,15]. Shin and Johnson defined life satisfaction as “a global assessment of a person’s quality of life according to his chosen criteria” [16]. In 1985, Diener et al. developed the Satisfaction with Life Scale (SWLS), with five statements providing an overall judgment of life, to measure the concept of life satisfaction [14,17]. The psychometric properties of the SWLS have been extensively examined in various populations [13,14] and in different cultures and nations [12,18]; translations are available and have been validated in numerous languages. Notably, Korea began to measure SWB in 2013. SWB is often included in population-based surveys, such as the Germany Socio-Economic Panel [1], the Gallup World Poll [19], and the Behavioral Risk Factor Surveillance System [9,19], because of the broad importance of SWB and its relevance to policy.

The OECD created the OECD Better Life Index, which comprises 11 topics considered essential to quality of life: housing, income, jobs, community, education, environment, governance, health, life satisfaction, safety, and work-life balance [15]. Using similar methodologies, the United Nations’ World Happiness Report describes happiness in each region of the world. Moreover, the specific factors that explain the variance in happiness in each country have been determined using regression analyses [15,20].

Although the number of studies of SWB has increased, a more comprehensive and multidimensional SWB assessment tool, one that is more relevant to policy, is still needed [1,15,18]. The tool should be simple and valid to examine long-term adaptation by tracking SWB changes with an annual evaluation or even a daily assessment [1,21].

The objective of this study was threefold. The first objective was to develop a new SWB assessment tool, the Subjective Well-Being Inventory (SWBI), that assesses individual self-reported SWB. The second objective was to evaluate the psychometric properties of the SWLS and SWBI in a nationally representative sample in Korea [18]. The third objective was to provide normative data for the SWLS and SWBI in the general Korean population for more detailed insights into Korean policy.

## 2. Materials and Methods

### 2.1. Subject Recruitment and Data Collection

This survey was conducted from March to May 2018 to obtain data from the general population in Korea. The survey followed the guidelines of the 2016 Korean Census and considered the age and sex strata in each of 17 major cities and local districts. Probability proportion-to-size sampling [22], a technique for selecting a representative national sample, was used to adjust the difference in probability between larger and smaller sampled groups.

Of the 4000 eligible people who were randomly selected to participate in this survey, 30% responded. The inclusion criterion was an age of at least 19 years, as the Korean Constitution considers individuals older than 19 as adults. In the end, 1200 people completed the semi-structured, self-reported questionnaire. Trained interviewers from World Research Co., Ltd., who specialize in performing surveys in Korea, communicated with the survey participants. The interviewers explained the purpose of the survey and the exact contents of the questionnaire to the participants in person during the survey. Before the main survey was conducted, a pilot test was performed to ensure that the cultural characteristics of Korea were reflected in the survey items and to examine the importance of other factors in terms of well-being. The pilot test was held for four days, and a total of 300 people participated. Written informed consent was obtained from all participants, and an explanation of the purpose of the study was provided. The entire study process was approved by the Institutional Review Board of Seoul National University Hospital (IRB No. 1804-024-934).

### 2.2. Measurements

The OECD Better Life Index is based on 11 essential topics in the areas of quality of life [23]. However, we added the concept of “interpersonal relationships” to one of the SWBI topics in the pilot study based on the OECD Better Life Index and various review articles [24,25,26,27]. Before conducting this study, we also collected opinions on well-being indicators by conducting a pilot test (online preliminary survey) of 300 people (30 in each of 10 gender and age groups [males and females aged 20–29, 30–39, 40–49, and 50–60 years]). The results of the pilot study confirmed that in addition to the original 11 topics related to well-being, “leisure” and “family” are essential to well-being among the Korean general public. Thus, a total of 14 topics were determined to form the SWBI: (1) education, (2) family, (3) health, (4) civic engagement, (5) life satisfaction, (6) income, (7) safety, (8) leisure, (9) work-life balance, (10) interpersonal relationships, (11) housing, (12) community, (13) job, and (14) environment. We added global well-being (GWB) to the SWBI as a measure of overall well-being. In this questionnaire, an 11-point Likert scale (from 0 = worst to 10 = best) was used to score the SWBI answer sheets.

The SWLS, which is designed to measure life satisfaction according to SWB [17], was included in the questionnaire. The five items of the SWLS were answered using a 7-point Likert scale, where 1 = strongly disagree and 7 = strongly agree [17]. Data on sociodemographic characteristics (age, sex, marriage, education level, religion, resident status, monthly income, and occupation) were also collected during the survey.

In addition to the SWBI and SWLS, the survey included validated questionnaires, such as the McGill Quality of Life Questionnaire (MQOL) [28] for SWB (especially for spiritual and social support) 41 and the Patient Health Questionnaire-9 (PHQ-9) for depression [29].

### 2.3. Statistical Analysis

Descriptive statistics were used to report demographic characteristics. In addition, the mean and standard deviation (SD) of the data for each topic (five items in the SWLS and 14 topics in the SWBI) were calculated to provide normative data categorized by age and sex. The SWLS scores were determined according to the survey scoring instructions [17]. Cronbach’s alpha and item-total correlation were used to determine the reliability of each of the topics in the SWBI and SWLS. Generally, the adequacy of the sample was confirmed by a Kaiser-Meyer-Olkin (KMO) index value of >0.8 and the value of Bartlett’s test of sphericity. Varimax orthogonal rotation was used in addition to the percentage of explained variance of the two indexes to implement an exploratory factor analysis and identify an eigenvalue.

Correlation analysis of the mean values of the SWBI and SWLS was also implemented. Prior to multiple logistic regression analysis, the following dummy variables were defined as a reference of each demographic characteristic: age (1 = 50 or older, 0 = under 50); sex (1 = female, 0 = male); marital status (1 = never married/divorced/separated/widowed, 0 = married); education level (1 = high school graduate or less, 0 = university graduate or more); religion (1 = having no religion, 0 = being religious); residence location (1 = metropolitan area; 2 = suburban area, 3 = rural area); income (1 = less than 4 million Korean won (KRW) of household monthly income, 0 = 4 million KRW or more of household monthly income [1 USD = 1130 KRW]); occupation (1 = employed, 0 = not employed); and scores of both the SWBI and SWLS models (1 = less than 7 points, 0 = 7 points or more). Using a stepwise selection technique of multiple logistic regression analysis, adjusted odds ratios (aORs) were calculated, and demographic factors of significance to the SWBI and SLWS were selected. The maximum likelihood method was used to calculate 95% confidence intervals (CIs). The SPSS statistical software package version 23.0 (SPSS Inc., Chicago, IL, USA) was used for all analyses.

## 3. Results

The demographic characteristics of the participants in this study are shown in Table 1. The mean and SD score and the reliability of both the SWLS and SWBI models are shown in Table 2. The SWLS had a Cronbach’s alpha of 0.89 and an item-total correlation of 0.710–0.752. The KMO index was 0.912, and Bartlett’s test of sphericity was chi-squared = 3342.12 (*p* < 0.001), with 68.98% of total explained variance and an eigenvalue of 3.449. Of the 14 topics comprising the SWBI, family (7.08 ± 1.42) and health (7.06 ± 1.42) status had the first and second highest mean scores, respectively, in the Korean general population. In contrast, income (6.40 ± 1.63) and environment (6.21 ± 1.85) status had the lowest mean scores. Cronbach’s alpha of internal consistency was 0.946, and each value of item-total correlation was in the range of 0.651–0.800. Exploratory factor analysis revealed a 59.32% total variance and a characteristic value (eigenvalue) of 8.305. The KMO index (0.958) showed the adequacy of the sample, and a high dependence among the 14 topics was indicated by Bartlett’s test of sphericity (chi-squared = 9185.32, *p* < 0.001).

As shown in Table 4, men had a lower total life satisfaction score than women on the SWLS, but a higher proportion of men than women indicated satisfaction. The 20 s and 30 s groups had the highest percentages of satisfaction irrespective of sex. Among men, the total SWLS was higher for younger participants, except that those aged 60 and above had a higher score than those in their 50s. In addition, in women, a similar trend was found, except that those in their 40 s had the highest total SWLS score.

The 14 topics in the SWBI were scored according to age group and sex. As seen in Table 4, “health” and “family” were rated as two of the highest scored items among men, and “interpersonal relationships” showed the highest score of importance followed by “health” and “family” among women. In addition, “environment” came in last for both men and women.

As shown in Table 5, two characteristics significantly associated with higher SLWS scores were younger age (aOR = 1.303, 95% CI = 1.003–1.694) and living in a rural area (aOR = 1.628, 95% CI = 1.280–2.070). These results were consistent with the SWBI, with sex, marital status, and income level being selected as additional significantly related variables. The results (Table 5) indicated a significant positive correlation between being single (aOR = 1.332, 95% CI = 1.039–1.708) or having a higher income (aOR = 1.299, 95% CI = 1.020–1.654) and higher SWBI scores.

## 4. Discussion

To the best of our knowledge, this is the first study to present SWLS data from a general Korean population sample. As conceptualized by Diener et al. [17], confirmatory factor analysis of SWLS data. To the best of our knowledge, this is the first study to present SWLS data from a general Korean population sample. As conceptualized by Diener et al. [17], confirmatory factor analysis of SWLS data showed very good psychometric properties with a unidimensional scale and high internal consistency (Cronbach’s alpha = 0.89). This result is in accordance with numerous previous studies of different ethnic groups of the general population [9,11,12,14,17]. Additionally, the SWBI shows good psychometric properties, with a unidimensional scale and high internal consistency, and could be a reliable measure of SWB in the Korean context. The data of the SWLS and SWBI can be used as reference data in future studies.

This study shows that age was significantly associated with the SWBI and SWLS scores. These findings are in line with an earlier study [30], but some studies showed that neither age nor sex was significantly associated with the SWBI or SWLS [12,31,32]. Marriage was associated with higher SWBI scores, which is consistent with recent research findings suggesting that marriage can boost SWB [1,33,34]. In contrast, with previous studies [30,35], people with higher educational levels did not show a higher level of life satisfaction and SWB. Some studies indicate that religious people experience greater life satisfaction than nonreligious people [36,37]. Therefore, more studies need to be performed with different groups and populations to generalize and corroborate these findings.

The results of this study suggest that income has a positively significant relationship with SWB but not with SWL. These findings are consistent with earlier studies showing a positive association between individuals’ incomes and their SWB [9,37,38]. It is believed that income allows people to fulfill most of their physical needs and even some of their psychological needs, such as respect, to a certain degree [9]. Conversely, a longitudinal study suggests that better SWB might influence higher income [39]. Unemployment has undeniable serious financial implications and likely reduces self-respect, social status, and confidence [37,40]. The negative impact of unemployment is not explained by the loss of income alone [1]; there is substantial evidence that job loss leads to lower levels of SWB [1,15]. However, this study showed that employment was not associated with SWL and SWB. Income has shown to directly benefit SWB in the Korean population more than employment does [37]. These findings need to be confirmed by further studies.

The validation data of the SWBI in national surveys show the potential utility of SWBI in research in other groups or contexts. In particular, given the significant association of the SWLS with SWBI, the use of the SWBI together with the SWLS might improve measurements to capture well-being [18,37]. National accounts of the SWLS and SWBI will provide a summary of diverse dimensions of well-being in Korean society. Two reviews show that SWB was correlated with good outcomes, such as productivity at work, good citizenship, parenting, social relationships, and lower levels of mortality from cancer and chronic disease [1,38,41,42]

This study has several limitations. First, the fact that we did not examine test–retest reliability and interobserver agreement are limitations of the current study. Second, it is uncertain whether a causal direction between income and SWB, as measured by the SWBI, can be confirmed. Policy leaders are skeptical of “soft” data. We need stronger evidence for the causal effects of SWB on income. The direction of a dynamic relationship could be confirmed in longitudinal studies with more rigorous data [1,37]. Finally, our study does not support the indication that having religious beliefs positively influences life satisfaction. Although we administered nationwide questionnaires to the general Korean population, the results were derived from relatively few questions-based entirely on our own concepts and assessed in a single study [37].

In conclusion, this study showed good psychometric properties of the SLWS and SWBI, which could be applicable to Korea Our results also provide valuable normative data for evaluating the SWLS and SWBI, but additional studies are required to support the usefulness of these parameters for additional social or political purposes.

## 5. Conclusions

In conclusion, this study showed good psychometric properties of the SLWS and SWBI, which could be applicable to Korea.

## Figures and Tables

**Table 1:** Demographics of the Study Participants.

Variables	N	%
**Sex**		
Male	592	49.3
Female	608	50.7
**Age (years)**		
< 50	655	54.6
≥ 50	545	45.4
**Marital Status**		
Married	884	73.7
Separated / Divorced / Widowed	51	4.2
Single	265	22.1
**Educational Level**		
≤ High School Graduates	661	55.1
> High School Graduates	539	44.9
**Religion**		
No	709	59.1
Yes	491	40.9
**Residence**		
Metropolitan Area	543	45.3
Suburban	592	49.3
Rural	65	5.4
**Monthly Income** (1 USD = 1110 KRW)		
<4 million KRW	651	54.3
≥4 million KRW	539	45.7
**Employment Status**		
Unemployed	360	30.0
Employed	840	70.0

**Table 2:** Mean, Standard Deviation (SD), Cronbach’s alpha, and Item-Total Correlation of Each Item in the Satisfaction with Life Scale and Subjective Well Being Index.

Item	Mean	SD	Cronbach’s alpha **	Item-total correlation
**Satisfaction with Life Scale**			0.886	
In most areas, my life is close to my ideal	4.40	1.06	0.855	0.752
The conditions of my life are excellent	4.50	1.02	0.862	0.724
I am satisfied with my life	4.57	1.04	0.861	0.728
So far, I have gotten the things that are important to me in life	4.33	1.05	0.863	0.718
If I were born again, I would change almost nothing in my life	4.05	1.19	0.867	0.710
* Characteristic value (eigenvalue)				3.449
* Percentage of explained variance				68.98%
**Subjective Well Being Index**			0.946	
Education	6.55	1.55	0.943	0.688
Family	7.06	1.42	0.944	0.684
Health	7.08	1.58	0.944	0.683
Civic Engagement	6.82	1.52	0.942	0.728
Life Satisfaction	6.81	1.44	0.941	0.800
Income	6.40	1.63	0.942	0.756
Safety	6.92	1.48	0.942	0.727
Leisure	6.50	1.58	0.943	0.716
Work-Life Balance	6.59	1.56	0.941	0.768
Interpersonal/Human Relationship	7.05	1.44	0.942	0.731
Housing	6.93	1.46	0.942	0.745
Community	6.72	1.41	0.942	0.766
Job	6.44	1.61	0.942	0.742
Environment	6.21	1.75	0.945	0.651
* Characteristic value (eigenvalue)	8.305			
* Percentage of explained variance	59.32%			
* Components’ matrix with varimax orthogonal rotation				
** Cronbach’s alpha value if an item is deleted.				

A significant positive correlation between the SWLS and SWBI (*r* = 0.593, *p* = 0.01) was found (Table 3). The correlations of GWB with the SWLS and SWBI were also found to be significant, with R-values of 0.580 and 0.820, respectively (*p* = 0.01). The SWLS, SWBI and GWB were also significantly correlated with subscales of the MQOL (*p* = 0.01). SWLS, SWBI, and GWB were also negatively correlated with the PHQ-9 (*p* = 0.01).

**Table 3:** Correlations among the mean values of the Satisfaction with Life Scale (SWLS), Subjective Well Being Index (SWBI), Global Well-Being (GWB), McGill Quality of Life (MQOL) and Patient Health Questionnaire-9 (PHQ-9).

Scale	SWLS	SWBI	GWB
SWLS	1		
SWBI	0.593 *	1	
GWB	0.580 *	0.820 *	1
MQOL, Social support	0.450 *	0.650 *	0.594 *
MQOL, Existential well-being	0.503 *	0.706 *	0.658 *
PHQ-9	−0.305 *	−0.435 *	−0.425 *

* *p* = 0.01.

**Table 4:** Crude Mean Scores of Subjective Well-Being and Satisfaction with Life by Sex and Age Groups.

Sex	All populations						Men						Women					
**Age (years)**	Total	20–29	30–39	40–49	50–59	≥ 60	Total	20–29	30–39	40–49	50–59	≥60	Total	20–29	30–39	40–49	50–59	≥60
**No (%)**	1200 (100.0)	194 (16.2)	212 (17.7)	249 (20.8)	239 (19.9)	306 (25.5)	592 (100.0)	101 (17.1)	109 (18.4)	125 (21.1)	121 (20.4)	136 (22.9)	608 (100.0)	93 (15.3)	103 (16.9)	124 (20.4)	118 (19.4)	170 (27.9)
SWLS *																		
1. In most ways, my life is close to my ideal.	4.40	4.50	4.51	4.36	4.28	4.38	4.41	4.39	4.59	4.27	4.29	4.54	4.38	4.62	4.43	4.44	4.27	4.26
2. The conditions of my life are excellent.	4.50	4.57	4.53	4.58	4.4	4.43	4.47	4.54	4.52	4.46	4.37	4.49	4.52	4.60	4.53	4.69	4.43	4.39
3. I am satisfied with my life.	4.57	4.69	4.62	4.57	4.53	4.51	4.55	4.71	4.64	4.44	4.40	4.57	4.60	4.67	4.59	4.69	4.67	4.45
4. So far, I have gotten the important things I want in life.	4.33	4.34	4.29	4.34	4.33	4.34	4.33	4.24	4.34	4.30	4.34	4.40	4.33	4.45	4.24	4.37	4.33	4.29
5. If I could live my life over, I would change almost nothing.	4.05	4.04	4.14	4.02	3.93	4.12	4.06	4.07	4.22	3.89	3.89	4.24	4.04	4.01	4.05	4.16	3.97	4.03
Total Score (0–35)	21.9	22.1	22.1	21.9	21.5	21.8	21.8	22.0	22.3	21.4	21.4	22.2	21.9	22.4	21.8	22.4	21.6	21.4
% of Satisfied†	54.8	59.8	55.7	55.4	51.1	53.3	55.7	59.4	58.7	53.6	49.6	58.1	53.8	60.2	52.4	57.3	52.5	49.4
SWBI **																		
Education	6.55	7.15	6.87	6.67	6.41	5.97	6.70	7.11	6.78	6.78	6.68	6.28	6.41	7.19	6.97	6.56	6.14	5.73
Family	7.06	7.45	7.23	7.01	7.00	6.77	7.12	7.46	7.24	6.99	7.03	6.98	7.00	7.44	7.21	7.03	6.97	6.61
Health	7.08	7.69	7.33	7.19	6.83	6.6	7.29	7.82	7.52	7.30	6.94	7.01	6.87	7.55	7.13	7.09	6.72	6.27
Civic Engagement	6.82	7.16	6.95	6.89	6.72	6.53	6.98	7.17	7.08	7.03	6.91	6.78	6.66	7.15	6.81	6.74	6.53	6.32
Life Satisfaction	6.81	7.23	6.92	6.91	6.72	6.47	6.86	7.14	6.91	6.84	6.81	6.66	6.77	7.33	6.93	6.98	6.63	6.32
Income	6.40	6.43	6.47	6.59	6.46	6.14	6.46	6.31	6.61	6.56	6.45	6.39	6.34	6.57	6.33	6.61	6.46	5.95
Safety	6.92	7.13	6.91	7.01	6.99	6.66	6.98	7.10	7.00	7.02	6.91	6.89	6.86	7.17	6.82	6.99	7.07	6.48
Leisure	6.50	6.92	6.61	6.47	6.39	6.27	6.55	6.94	6.70	6.39	6.40	6.43	6.45	6.89	6.52	6.56	6.38	6.15
Work-Life Balance	6.59	6.95	6.59	6.63	6.54	6.37	6.64	6.97	6.69	6.59	6.47	6.57	6.54	6.92	6.49	6.68	6.60	6.21
Interpersonal Relationship	7.05	7.30	7.08	7.06	6.98	6.90	7.08	7.28	7.13	7.09	6.95	7.02	7.01	7.33	7.04	7.02	7.02	6.80
Housing	6.93	7.25	6.92	6.94	6.90	6.76	6.98	7.33	6.95	6.86	6.93	6.89	6.89	7.16	6.87	7.01	6.87	6.66
Community	6.72	6.91	6.68	6.77	6.74	6.59	6.77	6.94	6.67	6.74	6.77	6.75	6.68	6.87	6.69	6.80	6.72	6.45
Job	6.44	6.64	6.53	6.50	6.49	6.16	6.61	6.65	6.82	6.65	6.54	6.43	6.28	6.62	6.22	6.35	6.45	5.95
Environment	6.21	6.24	6.22	6.29	6.19	6.13	6.35	6.46	6.36	6.28	6.19	6.46	6.07	6.01	6.08	6.30	6.19	5.86

Abbreviations: SWLS, Satisfaction with Life Scale; SWBI, Subjective Well Being Index. * The answers of 5 items of the SWBI were rated using a 7-point Likert scale (1 = strongly disagree and 7 = strongly agree). † >21 points (scores of slightly satisfied). ** The answers of 14 topics of the SWBI were rated using 11-point Likert scale (from 0 = worst to 10 = best).

**Table 5:** Multiple Logistic Regression Analysis of Demographic Factors with SWLS and SWBI *.

Demographic Factor	No (%)	SWLS		SWBI	
		aOR (95% CI)	*p*-Value	aOR (95% CI)	*p*-Value
Age					
<50	655 (54.6)	1.303 (1.003–1.694)	0.048	1.467 (1.119–1.923)	0.005
≥50	545 (45.4)	Ref		Ref	
Sex					
Male	592 (49.3)	N/S	N/S	1.332 (1.039–1.708)	0.024
Female	608 (50.7)	Ref		Ref	
Marital Status					
Married	884 (73.7)	N/S	N/S	1.507 (1.129–2.011)	0.005
Single	316 (26.3)	Ref		Ref	
Education Level					
≤High School Graduates	661 (55.1)	N/S	N/S	N/S	N/S
>High School Graduates	539 (44.9)	Ref		Ref	
Religion					
Yes	491 (40.9)	N/S	N/S	N/S	N/S
No	709 (59.1)	Ref		Ref	
Residential Area					
Rural	65 (5.4)	1.628 (1.280–2.070)	<0.001	2.300 (1.352–3.910)	0.002
Metropolitan/Suburban	1135 (94.6)	Ref		Ref	
Household Income					
≥4 million KRW	539 (45.7)	N/S	N/S	1.299 (1.020–1.654)	0.034
<4 million KRW	651 (54.3)	Ref		Ref	
Occupation Status					
Employed	840 (70.0)	N/S	N/S	N/S	N/S
Unemployed	360 (30.0)	Ref		Ref	

Abbreviations: SWLS, Satisfaction with Life Scale; SWBI, Subjective Well Being Index; aOR, adjusted odds ratio; CI, confidential interval. * SWLS score of 21 or more and SWBI average score of 7 or more were classified as satisfied.
